# Common variants in *LAMC1* confer risk for pelvic organ prolapse in Chinese population

**DOI:** 10.1186/s41065-020-00140-2

**Published:** 2020-07-07

**Authors:** Juan Chen, Lei Li, Jinghe Lang, Lan Zhu

**Affiliations:** Department of General Gynecology, Peking Union Medical College Hospital, Chinese Academy of Medical Sciences, Beijing, 100730 China

**Keywords:** Pelvic organ prolapse, Polymorphisms, SNP, *LAMC1*

## Abstract

**Background:**

Pelvic organ prolapse (POP) affects around 15% of postmenopausal women in China. Although it has been widely accepted that genetic variants could confer risk for POP, the genetic susceptibility variants remain largely unknown. Previous studies indicated that *LAMC1*, which encodes the laminin gamma 1 chain and is critical for extracellular matrix, might be a susceptibility gene for POP. The study is to test the correlation of common variants across the *LAMC1* gene with POP susceptibility in Chinese population.

**Methods:**

A total of 396 individuals, including 161 unrelated patients of POP and 235 healthy controls, were recruited. Ten SNPs, including rs20558, rs20563, rs10911193, rs6424889, rs10911241, rs3768617, rs12073936, rs729819, rs10911214 and rs869133, of *LAMC1*, were genotyped using standard Sanger sequencing. The UNPHASED program (version 3.1.5) was used to analyze the genotyping data for allelic and genotypic associations.

**Results:**

SNP rs10911241 was significantly associated with POP risk (χ^2^ = 10.70, *P* = 1.1 E-03). The minor allele (rs10911241-G) carriers exhibited an increased risk of the disease (OR = 1.71, 95% CI = 1.24–2.36).

**Conclusion:**

Association of *LAMC1* with POP risk in Chinese population strongly supported the involvement of *LAMC1* in POP development.

## Introduction

Pelvic organ prolapse (POP) is a major health concern for women in menopausal period, greatly impair overall quality of life (QoL) [1]. Recent epidemiology studies showed that symptomatic POP affected nearly 15% of postmenopausal women in China [2], and 6–19% of them undergo surgery for POP, while up to 29% undergo reoperation within 3 to 5 years [3]. Though many studies have been conducted to unravel the molecular basis of POP, the underlying pathophysiology mechanisms remain largely unknown.

Both environmental factors, including vaginal parity, advancing age, obesity, prior surgery and hormonal status, and genetic variants have been reported to contribute to increased risk of POP [4]. Twins study showed that genetic factors accounted for 43% susceptibility of POP [5]. There is increasing evidence that POP is familial clustering and heritable. The relative risks of POP were significantly elevated in first- and third-degree female relatives [6]. In addition, a systematic review and quantitative meta-analysis revealed that family history was one of the major risk factors for prolapse recurrence after reconstructive surgery [7]. Mechanistically, POP is biomechanical weakness of pelvic supportive tissues due to disturbance in connective tissue metabolism [8]. The connective tissue contains relatively few kinds of cells, most of which are fibroblasts, producing components of the extracellular matrix (ECM). Up till now, several genes of ECM have been reported to be associated with POP though association study and linkage analysis, such as *MMP-1*, *MMP-3*, *MMP-9* [9], *COLIA1* and *LAMC1* [10]. Among them, *LAMC1*, which encodes the laminin gamma 1 chain and is critical for ECM, has been investigated for association with POP in European and American populations. Nikolova et al. identifies one SNP, rs10911193, located in *LAMC1* in a study of familial prolapse [11]. However, the correlation of *LAMC1* with POP susceptibility was absent in several different populations [12–14]. Chen et al. performed a case-control study of 265 Caucasians and 146 African Americans, and reported 3 SNPs in *LAMC1*, namely rs10911193, rs20563 and rs20558 [12]. However, no significant SNPs of *LAMC1* were associated with advanced prolapse within ethnicities. Wu et al. genotyped 14 SNPs in *LAMC1* and failed to correlate these SNPs with POP risk among 239 POP patients and 197 healthy individuals in American populations [14]. In contrast to the extensive studies in European and American populations, whether *LAMC1* was associated with POP risk in Chinese population remains to be investigated.

In the present study, we hypothesized that common variants in *LAMC1* might contribute to POP in the Chinese population. To test our hypothesis, we genotyped three previously reported risk SNPs and seven tag SNPs, across *LAMC1*, and tested their association with POP susceptibility.

## Methods and materials

### Subjects

A total of 396 individuals, including 161 unrelated patients with POP and 235 controls without POP were recruited from our hospital, Beijing, China, between July 2012 and September 2017. All the subjects were of China origin geographically and Chinese population. Family history of each individual was investigated and none of them belonged to extended family. All patients were clinically diagnosed by a senior urogynecologist according to the criteria of the International Continence Society to determine the stage of POP (pelvic organ prolapse quantification, POP-Q), and all of the POP patients were stage III (139 cases) or IV (22 cases). Controls were from routine health check-up department in the same hospital. All the control group were postmenopausal, and none of them accepted hormone therapy and prolapse surgery in the previous years. All the controls were of stage 0 (189 controls) or I (46 controls). For both groups, individuals with chronic pelvic inflammatory diseases, endometriosis, gynecological malignancies or connective tissue diseases were excluded. As showed in Table 1.

**Table 1:** Demographic features of women with and without pelvic organ prolapse

Variables	Non-POP (***n*** = 235) No.(%)	POP (***n*** = 161) No.(%)	***P*** value
**Age**^**a**^**(y)**	59(56,65)	63(53,68)	0.513
**Body mass index**^**a**^	24.6(22.8, 27.2)	23.5(22.4,25.5)	0.041
**Menopausal years**^**a**^	8(5,12)	14(8,19)	< 0.001
**Gravity**^**a**^	2(1,2)	3(2,4)	< 0.001
**Parity**^**a**^	1(1,1)	2(1,2)	< 0.001
**Hypertension**^**b**^	53	39	0.699
**Diabetes**^**b**^	22	13	0.658

^a^ The data are presented as the medians (interquartile range), and the *P* value was calculated with the Mann-Whitney test

^b^The data are presented as n (%), and the *P* value was calculated with the chi-square test

All participants provided written informed consent. This study was approved by the Medical College Hospital Ethics Committee (project No. S-450).

### SNPs selection

There were 3 SNPs, rs20558, rs20563 and rs10911193, of *LAMC1* that have been investigated in previous studies. In the present study, we first genotyped these three SNPs and then selected Tag SNPs using the Haploview 4.2 program on the basis of Chinese in Beijing (CHB) population from the Hapmap database (http://www.hapmap.org/), for association study in our population (Table 2). A total of seven Tag SNPs (rs6424889, rs10911241, rs3768617, rs12073936, rs729819, rs10911214 and rs869133) with minor allele frequency (MAF) of ≥5% across the whole *LAMC1* locus were selected, covering 86% of the gene.

**Table 2:** 10 SNPs that were selected for genotyping

SNP	Position^**a**^	Alleles	Function	Exonic Function	AA Change
**rs20558**	183,125,412	T > C	exonic	missense	LAMC1:NM_002293:exon15:c.T2663C:p.L888P
**rs20563**	183,116,620	A > G	exonic	missense	LAMC1:NM_002293:exon7:c.A1372G:p.I458V
**rs10911193**	183,021,513	C > T	2 KB Upstream	.	.
**rs6424889**	183,122,661	G > C	intronic	.	.
**rs10911241**	183,094,001	A > G	intronic	.	.
**rs3768617**	183,123,365	C > T	intronic	.	.
**rs12073936**	183,092,755	T > G	intronic	.	.
**rs729819**	183,139,899	G > A	intronic	.	.
**rs10911214**	183,056,650	T > C	intronic	.	.
**rs869133**	183,100,146	G > C	intronic	.	.

*SNP* Single-nucleotide polymorphism, *AA* Amino acid, *L* Leucine, *P* Proline, *I* Isoleucine, *V* Valine

^a^Chromosomal positions are based on GRCh38 and all SNPs are located on Chromosome 1q25.3

### DNA sampling and genotyping

Genomic DNA was extracted from peripheral blood leukocytes using the FlexiGene DNA kit (QIAGEN, USA) strictly according to the manufacturer’s instructions.

SNP loci were genotyped by standard Sanger sequencing. Polymerase chain reaction (PCR) was performed using a 2 x PCR master mix (TIANGEN, China), 20 ng of genomic DNA and 5 pmol of forward and reverse primers, respectively. The cycling conditions involved an initial step at 95 °C for 10 min, followed by 30 cycles of denaturation at 95 °C for 30 s, annealing at 45–60 °C for 30 s and extension at 72 °C for 30 s. Replication of 5% samples was performed blind to the technical personnel and the concordance rate of the duplicated control samples was 100%. The genotype calling rate for each SNP was more than 95%.

### Statistical analysis

For the demographic data, median and interquartile range will be used to describe non-normally distributed continuous data, and frequency and percentages will be used to describe categorical variables. We used Mann-Whitney test significant association to compare continuous outcomes and chi-square test to compare dichotomous parameters between the two groups. Student t-test (two-tailed) was applied to compare age-distribution between the patient group and the control group and the χ^2^ goodness-of-fit test to estimate the Hardy–Weinberg equilibrium (HWE) for each SNP. The UNPHASED program (version 3.1.5) was used to analyze the genotyping data for allelic and genotypic associations [15]. To circumvent the inflation of false positive rates due to multiple testing [16], 100,00 permutations were performed using the UNPHASED program for the global null hypotheses in which all the odds ratios were equal. The significance level for all statistical tests was set at a corrected *P*-value of 0.05. A power calculation was conducted for a nonfamilial case-control association using the Power and Sample Size program. Given a sample size of 161 cases and 235 controls, we have greater than 73.2% power to detect an effect size of approximately 1.70 and higher.

## Results

The χ^2^ goodness-of-fit test showed that the genotypic distributions of rs20563 deviated from Hardy–Weinberg equilibrium (*P* < 0.05) in the control group, we therefore excluded this SNP in further study (Table 3). Associations of three SNPs, rs20558, rs20563 and rs10911193 within *LAMC1* with POP susceptibility have been reported previously, we therefore first investigated whether these three SNPs might also contribute to POP risk in our population. As shown in Table 4, none of the two previously reported SNPs, rs20558 or rs10911193, showed allelic or genotypic association with POP (all *P* > 0.05). Among the 7 tag SNPs, significant allelic association with POP was observed for rs10911241 (χ^2^ = 10.70, *P* = 1.1 E-03), which survived a strict correction with 10,000 permutations (global *P* = 7.1 E-03) (Table 4). The minor allele (rs10911241-G) carriers exhibited an increased risk of the disease (OR = 1.71, 95% CI = 1.24–2.36). Significant genotypic association was also detected for rs10911241 (χ^2^ = 14.98, df = 2, *P* = 5.6 E-04).

**Table 3:** The goodness-of-fit χ^2^ test for the Hardy-Weinberg equilibrium

SNP	Controls					Cases				
	1/1	1/2	2/2	χ2	*P*	1/1	1/2	2/2	χ2	*P*
rs20558	102	94	35	2.866	0.090	64	64	31	4.006	0.045
rs20563	73	96	65	7.458	0.006	53	62	44	7.530	0.006
rs10911193	192	39	4	1.430	0.232	123	31	6	4.471	0.034
rs6424889	95	93	37	2.95	0.086	72	57	31	9.019	0.003
rs10911241	140	80	10	0.11	0.736	82	53	25	9.308	0.002
rs3768617	81	113	34	0.283	0.595	59	77	20	0.438	0.508
rs12073936	188	42	3	0.140	0.708	126	31	4	1.471	0.225
rs729819	95	104	29	0.004	0.948	57	83	19	1.830	0.176
rs10911214	147	72	8	0.050	0.822	99	41	16	11.11	8.6 E-04
rs869133	202	23	2	2.018	0.155	139	19	1	0.155	0.693

“1” represents the major allele and “2” represents the minor allel

**Table 4:** Allelic and genotypic association of 9 SNPs in *LAMC1* gene with POP

SNP	Sample	*N*	Allele frequency (%)		OR (95% CI)	χ^2^	*P*	Genotype frequency (%)			χ^2^	*P*
rs20558			C	T				CC	CT	TT		
	POP	159	192 (60.4)	126 (39.6)	1.192 (0.888–1.601)	1.372	0.241	64 (40.3)	64 (40.3)	31 (19.4)	1.393	0.498
	CTR	231	298 (64.5)	164 (35.5)				102 (44.2)	94 (40.7)	35 (15.2)		
rs10911193			C	T				CC	CT	TT		
	POP	160	277 (86.6)	43 (13.4)	1.397 (0.899–2.170)	2.229	0.135	123 (76.9)	31 (19.4)	6 (3.8)	2.270	0.321
	CTR	235	423 (90.0)	47 (10.0)				192 (81.7)	39 (16.6)	4 (1.7)		
**rs6424889**			C	G				CC	CG	GG		
	POP	160	201 (62.8)	119 (37.2)	1.003 (0.746–1.350)	4.67 E-04	0.957	72 (45.0)	57 (35.6)	31 (19.4)	1.403	0.496
	CTR	225	283 (62.9)	167 (37.1)				95 (42.2)	93 (41.3)	37 (16.4)		
**rs10911241**			A	G				AA	AG	GG		
	POP	160	103 (32.2)	217 (67.8)	1.709 (1.238–2.359)	10.70	**1.1 E-03**	82 (51.3)	53 (33.1)	25 (15.6)	14.98	**5.6 E-04**^a^
	CTR	230	100 (21.7)	360 (78.3)				140 (60.9)	80 (34.8)	10 (4.3)		
**rs3768617**			C	T				CC	CT	TT		
	POP	156	117 (37.5)	195 (62.5)	0.912 (0.678–1.226)	0.540	0.912	59 (37.8)	77 (49.4)	20 (12.8)	0.423	0.809
	CTR	228	181 (39.7)	275 (60.3)				81 (35.5)	113 (49.6)	34 (14.9)		
**rs12073936**			T	G				TT	TG	GG		
	POP	161	283 (87.9)	39 (12.1)	1.200 (0.766–1.880)	0.636	0.425	126 (78.3)	31 (19.3)	4 (2.5)	0.916	0.633
	CTR	233	418 (89.7)	48 (10.3)				188 (80.7)	42 (18.0)	3 (1.3)		
**rs729819**			A	G				AA	AG	GG		
	POP	159	197 (61.9)	121 (38.1)	1.115 (0.829–1.500)	0.515	0.473	57 (35.8)	83 (52.2)	19 (11.9)	1.693	0.429
	CTR	228	294 (64.5)	162 (35.5)				95 (41.7)	104 (45.6)	29 (12.7)		
**rs10911214**			T	C				TT	TC	CC		
	POP	156	239 (76.6)	73 (23.4)	1.270 (0.895–1.804)	1.795	0.180	99 (63.5)	41 (26.3)	16 (10.3)	7.637	0.022
	CTR	227	366 (80.6)	88 (19.4)				147 (64.8)	72 (31.7)	8 (3.5)		
**rs869133**			G	C				GG	GC	CC		
	POP	159	297 (93.4)	21 (6.6)	1.118 (0.620–2.016)	0.138	0.710	139 (87.4)	19 (11.9)	1 (0.6)	0.386	0.824
	CTR	227	427 (94.1)	27 (5.9)				202 (89.0)	23 (10.1)	2 (0.9)		

*POP* Pelvic organ prolapse, *CTR* Control, *SNP* Single-nucleotide polymorphism, *CI* Confidence interval, *OR* Odds ratio

^a^Global *P*-value was 7.1 E-03 after 10,000 permutations

## Discussion

Pelvic floor dysfunction is a major health issue for older women, and genetic factors are considered to play an important role in the pathophysiology of this disorder. In the present study, we performed an association study to investigate the common variants of *LAMC1* in POP susceptibility. A total of 10 SNPs, including 3 SNPs reported previously and 7 tag SNPs across the *LAMC1* gene locus, were selected for analysis. Among them, SNP rs10911241 showed both significant allelic and genotypic association with POP.

*LAMC1* gene is a large gene spanning 122 Kb and containing 28 coding exons, which encodes the gamma-1 chain of laminin. Laminins are important extracellular matrix glycoproteins, which composed of three chains: alpha, beta and gamma, creating 15 different isoforms. Laminins are the major non-collagenous constituent of basement membranes. Laminins are known to be involved in a variety of cellular mechanisms such as regulation of cell adhesion, differentiation, and migration [17, 18]. The polymorphisms of *LAMC1* gene have been reported to be associated with colorectal cancer, premature ovarian failure and Mayer-Rokitansky-Kuster-Hauser syndrome (*MRKHS*) [15, 19, 20]. Together with laminin, many kinds of proteins can synergize to maintain the pelvic support organization, involving collagens (I, II and III) [16], *MMP* family menbers which could cleave fibrillar collagen and denature peptides [21], *LOXL1* [22] and so on. Based on the functions of these genes in POP development, we hypothesized that variants in these genes might be associated with POP risk.

For *LAMC1*, rs10911193 showed significant association with POP risk previously [11]. Supporting our results, rs376617 and rs12073836 were previously studied by Wu et al. [14] and neither one showed association with POP. The absence of significant association of the above mentioned SNPs and POP risk in our cohort of samples might be due to the following reasons. First, the number of samples in both previous studies and the present study was small, which may cause inconsistent results as a result of sampling error. Second, population stratification, which can largely be dealt with statistically due to the difference in allele frequencies among subpopulations, would play an important role [23].

In addition, we genotyped seven new tag SNPs, explored their association with POP, and found that SNP rs10911241 was significantly associated with POP in both allelic and genotypic manners. Notably, rs10911241 was in strong linkage disequilibrium (LD) with rs10911193 (*R*^*2*^ = 1) while in weak LD with rs20558 or rs20563 (*R*^*2*^ = 0.079) in Chinese Han populations, suggesting that rs10911193-rs10911241 locus may be a truly positive signal for POP risk. Surprisingly, rs10911193 only exhibited a trend of association with POP despite of strong LD between rs10911193 and rs10911241, which is largely due to limited sample size recruited in the current study. Another important issue in a case-control study is careful selection of the control subjects considering that environmental components (such as increased gravity, increased time in post-menopausal status, etc) may also confer risk for POP. We can’t exclude the possibility that difference of environmental factors between cases and controls may confound the association results. Moreover, we do not know the ideal method for phenotyping pelvic organ prolapse. This point highlights the importance for future research into how best to phenotype prolapse, as well as the other pelvic floor disorders, and the importance of genetic and genetic epidemiological research of pelvic floor disorders.

In summary, we repeated the association of three *LAMC1* SNPs and seven new tag SNPs with POP risk in Chinese population, and found SNP rs10911241 to be significantly associated with POP risk, further in support of the important functions of LAMC1 in POP development. Our results provided evidences for further investigation of *LAMC1* in the pathophysiology of prolapse.

## Data Availability

The datasets during and/or analysed during the current study available from the corresponding author on reasonable request.

## References

[1] Jelovsek JE, Maher C, Barber MD (2007). Pelvic organ prolapse. Lancet.

[2] Li Z, Xu T, Li Z, Gong J, Liu Q, Wang Y (2019). An epidemiologic study of pelvic organ prolapse in postmenopausal women: a population-based sample in China. Climacteric.

[3] Walter JE, Urogynaecology C (2011). Transvaginal mesh procedures for pelvic organ prolapse. J Obstet Gynaecol Can.

[4] Barber MD (2016). Pelvic organ prolapse. BMJ.

[5] Altman D, Forsman M, Falconer C, Lichtenstein P (2008). Genetic influence on stress urinary incontinence and pelvic organ prolapse. Eur Urol.

[6] Norton PA, Allen-Brady K, Cannon-Albright LA (2013). The familiality of pelvic organ prolapse in the Utah population database. Int Urogynecol J.

[7] Friedman T, Eslick GD, Dietz HP (2018). Risk factors for prolapse recurrence: systematic review and meta-analysis. Int Urogynecol J.

[8] Kerkhof MH, Hendriks L, Brolmann HA (2009). Changes in connective tissue in patients with pelvic organ prolapse--a review of the current literature. Int Urogynecol J Pelvic Floor Dysfunct.

[9] Skorupski P, Jankiewicz K, Miotla P, Marczak M, Kulik-Rechberger B, Rechberger T (2013). The polymorphisms of the MMP-1 and the MMP-3 genes and the risk of pelvic organ prolapse. Int Urogynecol J.

[10] Cartwright R, Kirby AC, Tikkinen KA, Mangera A, Thiagamoorthy G, Rajan P (2015). Systematic review and metaanalysis of genetic association studies of urinary symptoms and prolapse in women. Am J Obstet Gynecol.

[11] Nikolova G, Lee H, Berkovitz S, Nelson S, Sinsheimer J, Vilain E (2007). Sequence variant in the laminin gamma1 (LAMC1) gene associated with familial pelvic organ prolapse. Hum Genet.

[12] Chen C, Hill LD, Schubert CM, Strauss JF, Matthews CA (2010). Is laminin gamma-1 a candidate gene for advanced pelvic organ prolapse?. Am J Obstet Gynecol.

[13] Nakad B, Fares F, Azzam N, Feiner B, Zilberlicht A, Abramov Y (2017). Estrogen receptor and laminin genetic polymorphism among women with pelvic organ prolapse. Taiwan J Obstet Gynecol.

[14] Wu JM, Visco AG, Grass EA, Craig DM, Fulton RG, Haynes C (2012). Comprehensive analysis of LAMC1 genetic variants in advanced pelvic organ prolapse. Am J Obstet Gynecol.

[15] Ravel C, Bashamboo A, Bignon-Topalovic J, Siffroi JP, McElreavey K, Darai E (2012). Polymorphisms in DLGH1 and LAMC1 in Mayer-Rokitansky-Kuster-Hauser syndrome. Reprod BioMed Online.

[16] Lince SL, van Kempen LC, Dijkstra JR, IntHout J, Vierhout ME, Kluivers KB (2014). Collagen type III alpha 1 polymorphism (rs1800255, COL3A1 2209 G>a) assessed with high-resolution melting analysis is not associated with pelvic organ prolapse in the Dutch population. Int Urogynecol J.

[17] Turck N, Gross I, Gendry P, Stutzmann J, Freund JN, Kedinger M (2005). Laminin isoforms: biological roles and effects on the intracellular distribution of nuclear proteins in intestinal epithelial cells. Exp Cell Res.

[18] Pouliot N, Saunders NA, Kaur P (2002). Laminin 10/11: an alternative adhesive ligand for epidermal keratinocytes with a functional role in promoting proliferation and migration. Exp Dermatol.

[19] Lou J, Gong J, Ke J, Tian J, Zhang Y, Li J (2017). A functional polymorphism located at transcription factor binding sites, rs6695837 near LAMC1 gene, confers risk of colorectal cancer in Chinese populations. Carcinogenesis.

[20] Pyun JA, Cha DH, Kwack K (2012). LAMC1 gene is associated with premature ovarian failure. Maturitas.

[21] Ferrari MM, Rossi G, Biondi ML, Vigano P, Dell'utri C, Meschia M (2012). Type I collagen and matrix metalloproteinase 1, 3 and 9 gene polymorphisms in the predisposition to pelvic organ prolapse. Arch Gynecol Obstet.

[22] Neupane R, Sadeghi Z, Fu R, Hagstrom SA, Moore CK, Daneshgari F (2014). Mutation screen of LOXL1 in patients with female pelvic organ prolapse. Female Pelvic Med Reconstr Surg.

[23] Burmeister M, McInnis MG, Zollner S (2008). Psychiatric genetics: progress amid controversy. Nat Rev Genet.

